# The Role of Metallurgical Features in the Microbially Influenced Corrosion of Carbon Steel: A Critical Review

**DOI:** 10.3390/microorganisms12050892

**Published:** 2024-04-29

**Authors:** Muhammad Awais Javed, Nicolò Ivanovich, Elena Messinese, Ruiliang Liu, Solange E. Astorga, Yee Phan Yeo, Sridhar Idapalapati, Federico M. Lauro, Scott A. Wade

**Affiliations:** 1 School of Science, Computing and Engineering Technologies, Swinburne University of Technology, Melbourne, VIC 3122, Australia; swade@swin.edu.au; 2 Asian School of the Environment, Nanyang Technological University, 62 Nanyang Drive, Singapore 637459, Singapore; nicolo001@e.ntu.edu.sg; 3 Department of Chemistry, Materials and Chemical Engineering “Giulio Natta”, Politecnico di Milano, Via Luigi Mancinelli, 7, 20131 Milan, Italy; elena.messinese@polimi.it; 4 Singapore Centre for Environmental Life Sciences Engineering, Nanyang Technological University, 60 Nanyang Drive, Singapore 637751, Singapore; ruiliang.liu@ntu.edu.sg (R.L.); solange.astorga@ntu.edu.sg (S.E.A.); yeephan001@e.ntu.edu.sg (Y.P.Y.); 5 Curtin Corrosion Centre, Faculty of Science and Engineering, Western Australia School of Mines (WASM), Curtin University, Perth, WA 6102, Australia; 6 School of Mechanical and Aerospace Engineering, Nanyang Technological University, 50 Nanyang Avenue, Singapore 639798, Singapore; msridhar@ntu.edu.sg; 7 Nanyang Environment & Water Research Institute (NEWRI), Nanyang Technological University, Cleantech ONE, 1 Cleantech Loop, Singapore 637141, Singapore

**Keywords:** carbon steel, grain size, inclusions, metallurgy, microbially influenced corrosion, microorganisms, microstructure

## Abstract

Microbially influenced corrosion (MIC) is a potentially critical degradation mechanism for a wide range of materials exposed to environments that contain relevant microorganisms. The likelihood and rate of MIC are affected by microbiological, chemical, and metallurgical factors; hence, the understanding of the mechanisms involved, verification of the presence of MIC, and the development of mitigation methods require a multidisciplinary approach. Much of the recent focus in MIC research has been on the microbiological and chemical aspects, with less attention given to metallurgical attributes. Here, we address this knowledge gap by providing a critical synthesis of the literature on the metallurgical aspects of MIC of carbon steel, a material frequently associated with MIC failures and widely used in construction and infrastructure globally. The article begins by introducing the process of MIC, then progresses to explore the complexities of various metallurgical factors relevant to MIC in carbon steel. These factors include chemical composition, grain size, grain boundaries, microstructural phases, inclusions, and welds, highlighting their potential influence on MIC processes. This review systematically presents key discoveries, trends, and the limitations of prior research, offering some novel insights into the impact of metallurgical factors on MIC, particularly for the benefit of those already familiar with other aspects of MIC. The article concludes with recommendations for documenting metallurgical data in MIC research. An appreciation of relevant metallurgical attributes is essential for a critical assessment of a material’s vulnerability to MIC to advance research practices and to broaden the collective knowledge in this rapidly evolving area of study.

## 1. Introduction

Carbon steels are the most widely used metals in the world, with approximately 2 billion tonnes produced in 2021 [1]. They can be mass-produced at a cost-effective rate and provide mechanical properties that are useful for a broad range of applications. For example, in construction and infrastructure, carbon steels are essential for building frameworks, reinforcing structures, and creating pipelines. Building and infrastructure make up about 50% of carbon steel demand [1]. Additionally, carbon steels are crucial in the manufacturing of storage tanks, ship hulls, sheet piling, and various support components vital for marine infrastructure. The cost of dealing with corrosion of carbon steels is a significant portion of the total cost of corrosion, which was estimated to be USD 2.5 trillion overall in 2013 [2].

A key issue with carbon steel, however, is degradation due to corrosion. Unprotected carbon steel is susceptible to many different corrosion mechanisms, and significant efforts and associated costs are devoted to corrosion prevention both in original equipment manufacturing (OEM) and maintenance, repair, and overhaul (MRO) operations. One of the lesser known but very important types of corrosion that affects carbon steels is microbially influenced corrosion (MIC). MIC can be defined as direct or indirect changes to corrosion due to the presence and/or metabolic activity of microorganisms [3,4]. A variety of microorganisms, including Bacteria, Archaea, and Eukarya, have been associated with MIC, with sulfate-reducing bacteria among the most widely recognized contributors [5,6,7]. The degradation and failure of carbon steel-based components/infrastructure due to MIC have been widely reported as responsible for costly environmental spills and a major health and safety issue [8].

Ferrous alloys are materials that primarily contain iron, with small amounts of other elements to tailor desired properties. Carbon steel, typically defined as being composed of iron and <2% alloying elements, is one of the most common types of ferrous metal (making up ~90% of the steel shipped in the United States) [9]. By varying the specific alloy content and manufacturing/processing processes, the properties of carbon steel can be altered for a particular application (e.g., increased tensile strength). For instance, the addition of chromium to carbon steel can enhance its corrosion resistance, making it suitable for use in aggressive environments such as marine applications. Similarly, manufacturing processes such as heat treatment can alter the microstructure of the steel, affecting its mechanical properties and corrosion resistance. Quenching and tempering, for example, can improve the strength and toughness of carbon steel; however, if not performed correctly, they can lead to the formation of brittle phases that increase susceptibility to corrosion. These changes to alloying and manufacturing processes fundamentally affect the microstructure of the steel, in addition to causing the presence of impurities and other defects. These, in turn, affect the rate, mechanism, and manifestation of, as well as the susceptibility to, corrosion [10]. Hence, work has been undertaken to produce steels that are more resistant to atmospheric corrosion [11] for marine applications (e.g., ASTM A690), as well as resistant to MIC [12].

This review is the first to address a gap in the research on MIC by assessing how the metallurgical properties of carbon steels affect MIC. The article begins by exploring microbial attachment and biofilm formation. It then delves into the fundamental mechanisms of MIC, followed by a critical re-analysis of the impacts of composition, microstructure, and inclusions. The discussion extends to examining the effects of welding on carbon steel in the context of MIC. Through this comprehensive review, our goal is to uncover fundamental observations and trends that shed light on the interactions between microorganisms and carbon steel, a material of immense significance in contemporary society. This could potentially lead to the development of strategies for mitigating carbon steel degradation caused by MIC. Additionally, the paper aims to provide insights into the current challenges associated with reporting and testing methods currently employed in MIC research involving carbon steel, along with suggestions for enhancing these practices.

In this study, we employed a systematic approach to select references for our review. First, we identified key topics related to MIC and the metallurgical properties of carbon steels. We then conducted a comprehensive literature search using databases such as Scopus, PubMed, Web of Science, and Google Scholar, with keywords related to MIC, carbon steel, metallurgy, and corrosion. Based on predefined selection criteria, we screened the search results to identify relevant references for inclusion in the review. These criteria included relevance to the topic, publication date, and credibility of the source. We extracted relevant data from the selected references, including information on metallurgical properties, MIC mechanisms, and experimental findings. The extracted data were analysed to identify trends, patterns, and gaps in the literature and synthesized to develop a comprehensive understanding of the topic. Finally, we compiled a list of selected references, created figures and tables to visually represent key findings, and conducted a final review to ensure that the selected references effectively supported the arguments and conclusions of the paper. This systematic approach ensured that our review was based on a rigorous and transparent methodology, enhancing the originality and credibility of our findings.

## 2. Overview of Interactions between Microorganisms and Metal Surfaces

This section provides a brief background of the initial attachment of microorganisms to surfaces and some of the material degradation processes that microorganisms can cause. It is important to note that while this paper is focused on the interactions with carbon steels, microorganisms attach to and can degrade a wide range of metals and other material types, such as plastic polymers, concrete, and ceramics. This section is not intended to be a detailed review but an introduction to key concepts and includes references with further detailed information that the reader can refer to as needed.

### 2.1. Initial Attachment of Microorganisms and Biofilm Formations

Microorganisms in nature live predominantly in complex communities and often adopt a matrix-enveloped sessile lifestyle known as biofilm. They colonize almost all known surfaces, from rocks, sediments, and animal guts to engineered materials such as plastic and steel [13,14,15,16,17]. In contrast to their free-living counterparts (known as planktonic cells), biofilm cells are typically more physiologically distinct and highly resistant to harsh environmental conditions, which greatly enhances their survival [18,19,20,21,22]. Biofilms are important participants in various biogeochemical and biotechnological processes, but at the same time, they are also involved in numerous activities that can lead to adverse economic impacts, such as biofouling and MIC [14,23,24,25,26].

Research into the model microorganism for biofilm formation, *Pseudomonas aeruginosa*, revealed a conceptual biofilm lifecycle that can be divided into five stages, namely (i) reversible attachment, (ii) irreversible attachment, (iii) extracellular polymeric substance (EPS) production/microcolony formation, (iv) maturation, and (v) dispersal [27,28,29,30]. The initial attachment phase, when the free-living planktonic cells encounter a surface, is typically temporary, reversible, and affected by the net attraction and repulsion of physical forces such as van der Waals forces and electrostatic interaction [27,31,32]. A range of other important chemical, mechanical, and biological factors such as chemotaxis (the movement of an organism towards or way from a chemical stimulus) [33,34], surface roughness [35], and surface-conditioning films [36,37] can also affect attachment.

Ultimately, over time, a biofilm is developed with a complex architecture, including channels for nutrient exchange [38,39]. While the in vitro biofilm model is generally accepted, the process of development and the consequent structure of biofilms in the real-world environment are often far more complex and dynamic [28]. They are influenced not just by environmental conditions but also by interactions and biological processes within the biofilm. It is therefore crucial to consider an environmental biofilm as a microbial consortium with distinct characteristics when evaluating its potential impact on the engineered materials used in such environments. The attachment of microbes can be influenced by the composition and diversity of the microbial community. This is because various species may have distinct attachment mechanisms and preferences for specific surface types [16,28]. It is important to take this into consideration when considering the type of laboratory model used for an MIC study and how it might influence the subsequent results [3].

### 2.2. Mechanisms of MIC of Steel 

MIC is a complex phenomenon that involves intricate and interdependent processes and thermodynamic reactions driven by interactions between microorganisms, metals, and the environment. Since early reports at the start of the 20th century [40], several MIC mechanisms have been proposed, focusing on specific groups of microorganisms (i.e., iron oxidizers, manganese oxidizers, and sulfate reducers), localized microenvironments (i.e., aerobic and anaerobic environments), and chemical reactions. Biofilm formation often drives these mechanisms, as microorganisms regularly attach to metallic surfaces and can subsequently modify chemical characteristics, creating aerobic and anaerobic areas where MIC can occur [41]. While there have been some advances in understanding, the complexity of biofilms and MIC has yet to be completely untangled.

In an abiotic aerobic aqueous environment, iron (Fe^0^), the major component of steel alloys, is oxidized to Fe^2+^ through an anodic reaction, while oxygen is reduced in a cathodic reaction. The activity of microorganisms attached to a metallic surface can disrupt this equilibrium, increasing, or (in some cases) decreasing the rate of one or both reactions. For instance, iron-oxidizing bacteria (IOB) use Fe^2+^ as an electron donor for oxygen reduction [42,43], promoting the abiotic dissolution of iron. Similarly, manganese-oxidizing bacteria (MOB) form MnO_2_ as result of Mn^2+^ oxidation. Such deposits act as cathodic sites and are reduced by the electrons released during the iron-dissolution step [8]. Additionally, the attachment and growth of microorganisms in the form of a biofilm on the metal surface, as explained in Section 2.1, can give rise to the formation of anaerobic microenvironments as a result of oxygen consumption in aerobic respiration. The difference in oxygen concentration between the anaerobic area and the surrounding aerobic environment creates an aeration cell, generating an electrical current (and, hence, corrosion) with electrons flowing from the anode to the cathode [44]. The formation of anaerobic microenvironments can also facilitate the proliferation of other corrosive anaerobic microorganisms [45,46].

A significant focus of research on MIC has been on the accelerated corrosion caused by anaerobic microorganisms such as sulfate-reducing bacteria and archaea. While there have been various names for the proposed corrosion mechanisms involved, they are typically divided between those involving electrical processes (EMIC) and those driven by chemical/metabolite processes (CMIC) [6,47]. Laboratory experiments have shown that the degradation mechanism taking place will depend on the material being studied, with carbon steel corrosion typically being driven by EMIC [47]. Other work suggests that a number of different electrical transfer processes can be affected by the presence of electron shuttles such as H_2_ and flavins and by changes in the levels of carbon sources present [48,49]. This emphasizes the importance of both careful planning and interpretation of MIC laboratory experiments, as their design can greatly influence the obtained results.

In addition to sulfate reducers, another category of microorganisms being increasingly studied in relation to MIC are methanogens. Methanogens, microorganisms that produce methane as a metabolic byproduct, have been identified in a variety of corrosion-relevant environments such as oil and gas pipelines and storage tanks [50,51] and metal sheet piles [52]. Various laboratory tests have shown significantly increased corrosion rates in the presence of specific methanogens, such as *Methanobacterium* IM1 [53], although other work has indicated that the composition of the culture medium used in the test and energy source starvation can influence the rate of corrosion [54]. Finally, studies indicate that hydrogenases from certain methanogens are linked to accelerated corrosion and that biomarkers for such hydrogenases may provide a means to monitor MIC [51].

While experiments using pure cultures and model organisms are essential for understanding the core mechanisms of MIC, care should be taken when extrapolating these studies to the corrosion processes associated with complex, multispecies biofilms prevalent in natural environments. It is also worth highlighting that, in addition to corrosion acceleration, microorganisms can also be involved in corrosion inhibition [55,56,57], an effect that is often dependent on the growth conditions or growth media [56]. Moreover, the effectiveness of a variety of microbially derived natural products and microbial processes in corrosion inhibition has been reported [58] and might lead to the development of engineered biofilms with anticorrosive or corrosion-protective properties. 

Various coatings like polyurethanes, epoxy resins, and others can also be used to protect metals from corrosion, including MIC, by isolating them from the electrolyte [59]. These coatings can be modified to enhance their performance and resist biodegradation, ultimately controlling microbial fouling and preventing localized corrosion. 

It is worth mentioning here that the environment plays a crucial role in MIC and corrosion prevention [56,59]. Factors such as temperature, pH levels, humidity, and the presence of specific microorganisms can significantly impact the rate and extent of corrosion. Understanding these influences is key to implementing effective corrosion prevention strategies, which may include selecting appropriate coatings, adjusting environmental conditions, or using inhibitors to mitigate corrosion risks [59].

## 3. Steel: Composition, Microstructure and MIC

### 3.1. Steel Composition and MIC

This section discusses the various systems used to categorize carbon and low-alloy steels, describes how alloying elements affect steel properties and characteristics, and reviews MIC studies conducted on different grades of carbon and low-alloy steels.

#### 3.1.1. Classification of Steel Based on Chemical Composition

**Figure 1:**
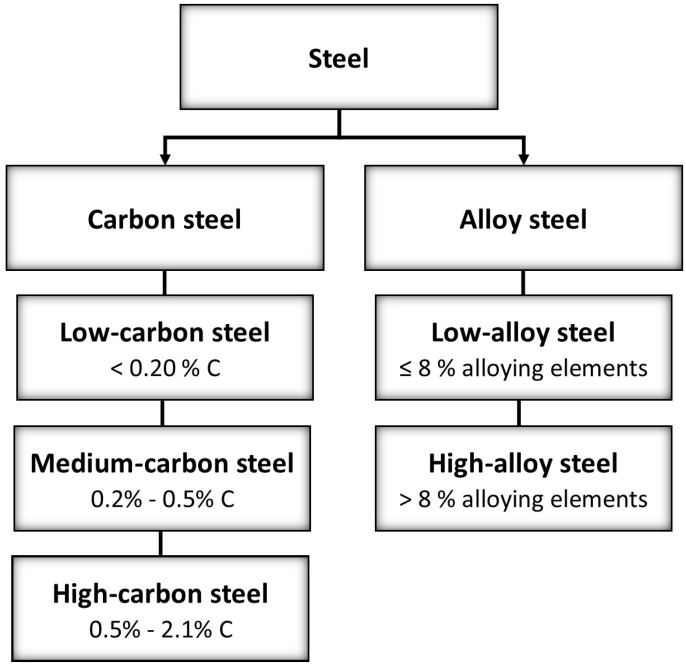
Classification of steel types based on chemical composition (with chemical compositions sourced from the *ASM Handbook* [9]).

Steel can be classified into different categories based on its chemical composition, including carbon steel, low-alloys steel, and high-alloy steel (Figure 1).

#### 3.1.2. Classification Based on Designation System

Designation systems have been developed by the steel industry to allow for the identification of the grade (chemical composition), type (deoxidation practice), and class (other characteristics such as mechanical properties) of a steel. In this section, we provide some examples of the popular steel designation systems [60,61].

The American Society for Testing and Materials (ASTM) classifications are a commonly used standard among steel producers, specifiers, and fabricators. The ASTM designation system for metals consists of a letter (often “A” for ferrous metals) followed by a numerically assigned value. For instance, the ASTM A36 designation represents a structural carbon steel alloy [60,61].

The American Iron and Steel Institute (AISI) and the Society of Automotive Engineers (SAE) have developed other well-used systems for classifying steels based on their standard chemical compositions. The AISI/SAE system uses a four-digit code, with the first two digits indicating the major alloying elements and the last two digits indicating the carbon content [60]. Carbon steels fall under the 1xxx group in the AISI system and can be further categorised into different subgroups based on specific properties, as listed in Table 1 [60,61,62]. Plain carbon steel with max 1% manganese is represented by 10xx. The second digit in each series denotes significant elements influencing steel properties. For instance, in 1018 steel, the “0” in the 10xx series signals the absence of major secondary elements like sulfur. The last two digits indicate the carbon content, such as 0.18% carbon in 1018 steel. In some cases, additional letters are added to the code between the second and third digits to denote other characteristics, such as “B” for 0.0005% to 0.003% boron alloying addition and “L” for 0.15% to 0.35% lead [60].

Finally, the unified numbering system (UNS) uses an alphanumeric code to designate individual alloys [61]. For carbon steel, the code consists of the letter “G” followed by the four numbers of AISI/SAE designation system, usually followed by “0” [61]. Likewise, AISI/SAE 1018 carbon steel would have a UNS designation of UNS G10180.

#### 3.1.3. Effects of Different Alloying Additions on Steel Properties

Pure metals are often intrinsically soft, and solution strengthening is one of the mechanisms to improve both physical and mechanical properties [63]. For example, iron, as a fundamental metal, is alloyed with carbon (up to a maximum solubility of 2%) to produce steel. The addition of carbon has a major effect on the strength and hardness of steel. Alloying elements play a crucial role in determining the properties of steel. By adding different elements to the iron–carbon matrix, the composition and microstructure of steel can be modified, resulting in a wide range of properties tailored to specific applications. Some common alloying elements and their effects on steel properties are provided in Table 2. It is important to note that these are just a few examples of alloying elements and their effects on steel properties. Different combinations and concentrations of these elements, along with other factors like heat treatment, can further modify the properties of steel to meet specific requirements for various applications.

#### 3.1.4. Alloying Addition in Steel and MIC

Considerable research has been conducted on steel composition and susceptibility to MIC [64,65,66,67]. For example, the study conducted by Gubner [64] investigated the effect of steel composition on MIC in marine environments. The research compared the MIC performance of marine-grade steels through field trials, focusing on the corrosion behaviour of low-alloy steels as an alternative sheet-piling material. The study exposed Grade 43A carbon steel, as well as two low-alloy steels—one containing alloying elements such as Cr 0.9%, Cu 0.29%, and Ni 0.23%, and the other containing Cr 0.56% and Si 1.35%—at Camber Dock, Portsmouth, UK, for up to 32 months. The study found that despite the presence of alloying elements intended to improve corrosion resistance, such as Cr, Cu, Ni, and Si, the bacterial colonization of all tested materials was rapid, with no statistically significant differences observed between the alloys. This suggests that the reported bactericidal effect of elements like chromium, copper, and nickel was not detected at the chosen concentrations. Overall, the research indicated that the use of low-alloy steels with these alloying elements did not significantly enhance the corrosion resistance of piling materials in marine environments.

In another study, Refait et al. [66] studied the role of Al (0.4 to 0.8%) and Cr (0.75 to 1.5%) alloying elements in the corrosion resistance of steel in natural seawater. They found that the presence of Al and Cr in low-alloy steel proved beneficial in natural seawater, where improved resistance to corrosion was observed after 6.5 months of immersion. This improvement was most notable in coupons without mill scale, indicating that the protective corrosion product layer forms spontaneously in seawater. Alloying elements Al and Cr were suggested to promote the formation of a more compact and adherent γ-FeOOH outer layer, enhancing the overall corrosion resistance of the steel. Additionally, it has been suggested that this particular steel composition exhibits enhanced resistance to MIC [68].

The influences of common alloying elements found in steel on the susceptibility of the individual steel type to MIC are summarised in Table 3. Further discussion on the impact of each alloying addition is provided as follows:

Carbon (C)—MIC is known to be influenced by the composition of steel, with carbon content playing a crucial role in determining the extent of MIC [67,69]. Alicia et al. [70] investigated the effect of sulfate-reducing bacteria (SRB) on the corrosion of different steel alloys (A179, A516-70, and A106-B) in industrial cooling water systems and found a direct correlation between carbon content of steel and SRB-induced MIC. Javed et al. [67] investigated the effects of varying carbon contents across different steel grades (1010, 1020, 1030, and 1045) on bacterial attachment and MIC. The results indicate that bacterial attachment decreased as carbon content increased, whereas the corrosion rates showed a linear increase with carbon content. In contrast, Cai et al. [71] studied corrosion behaviours of Q235, X65, X70, and X80 steels in a soil solution with a mixed SRB culture and found no direct relation between MIC and carbon content across different steel grades. They found that the MIC rate followed the order of X70 > Q235 > X65 > X80, whereas the carbon content increased in the order of Q235 > X70/X65 > X80. Similarly, Ashton et al. [72] examined the corrosion rates of six different carbon steel alloys (K1878, K1063, S2684, K795, V2170, and V2179) in the presence of *Escherichia coli* bacteria. They observed that the presence of *E. coli* increased the corrosion rates of the tested steel alloys without any correlation between the carbon content and MIC. 

The potential impact of carbon content on steel MIC might not be apparent in the latter two studies due to possible masking effects from microstructural variations, such as differences in grain size across the various types of steel employed. These microstructural variations play a significant role in steel MIC, as explained later in Section 3.2. Additional research is necessary to unravel the influence of grain size and carbon content in steel on its vulnerability to MIC.

Copper (Cu)—Although copper and its alloys are often used due to their antimicrobial properties, metals produced with copper as a major alloy are still vulnerable to MIC [73,74,75]. Examining the role of copper alloying in steel in the context of MIC resistance provides an intriguing insight into how steel reacts to environmental factors. Gino et al. [76] found that copper alloying within the range of 0.08 wt.% to 0.44 wt.% does not significantly impact MIC susceptibility for API 5LX steel when exposed to a natural consortium of bacteria and fungi. In contrast, Mansouri et al. [77] reported that positive corrosion inhibition arises from the addition of copper (0.002–0.29 wt.%). Shi et al. [78] observed that Cu-alloyed pipeline steel X80 exhibited effective antimicrobial behaviour against *E. coli*, *Staphylococcus aureus*, and SRB. Additionally, they measured reduced pit depths in X80 steel alloyed with 1.0 wt.% Cu [79]. Ke et al. [80] also reported similar beneficial effects of Cu alloying on X80 steel. Wu et al. [81] found that corrosion rates in different mooring-chain steels varied based on Cu content in the following order: BR5 (no Cu) < BR5CuH (0.8 wt % Cu) < BR5CuL (0.4 wt% Cu). They suggested that the introduction of Cu did not have a major impact on the quantity of microorganisms attached to the surface of the material. However, it did modify the diversity, richness, and structure of the microbial community due to the relative tolerance of certain species to Cu. The work of Wang et al. [82] also showed that Cu content in high-strength, low-alloy (HSLA) steels reduced MIC by *Halomonas titanicae* under anaerobic conditions. More recently, Zhong et al. [83] developed Cu-modified AISI 8630 steels with 0.4 wt% Cu, which showed significant improvement in the corrosion resistance of steel in a marine environment containing *Pseudomonas aeruginosa*. The enhanced corrosion resistance was shown to be due to the formation of a duplex oxide film on steel surfaces containing Cu_2_O and CuO.

Sulfur (S)—The presence of sulfur in steel is frequently associated with the existence of inclusions. Numerous studies have demonstrated that elevated levels of impurity elements, such as sulfur, correlate with increased susceptibility of alloyed steel to MIC [76,84,85,86,87]. The role of inclusions in MIC will be discussed in Section 3.4.

Cerium (Ce)—Cerium alloying content is believed to enhance resistance to MIC in low-alloy steels. Fong et al. [86] found that 8630 steel containing minor cerium content (0.01 wt.%) showed increased MIC resistance. Similar findings were reported in other studies. Gino et al. [83] found that alloys with minor cerium alloying (0.015 wt.%) exhibited lower corrosion current density values in natural consortia of bacteria and fungi, indicating reduced susceptibility to MIC compared to pristine alloys. Similarly, Walsh et al. [84] also indicated that enriched cerium alloying content decreased MIC susceptibility in 8630 steel welds.

Chromium (Cr)—Chromium plays a pivotal role as an alloying element in steel due to its ability to form a passive oxide film on the steel surface, which can serve as an effective barrier against corrosive agents, including microorganisms [88,89,90,91]. For instance, Nesterova et al. [92] explored the MIC resistance of pipeline steel with varied chromium contents in the presence of the SRB species *Desulfovibrio*. They found that increased chromium content (up to 5%) in steel impedes bacterial activity and the formation of SRB biofilm on the steel surface, consequently leading to a lower MIC rate.

Nickel (Ni)—Another vital alloying element in steel is nickel, which stabilises FCC austenite phases. Wang et al. [82] studied the impact of nickel alloying on the MIC behaviour of high-strength, low-alloy steel (HSLA) when exposed to *H. titanicae* under anaerobic conditions. Their findings indicated that nickel alloying enhanced the resistance of HSLA to MIC by forming nickel-rich oxide layers, such as NiFe_2_O_4_, which inhibited SRB adhesion and subsequent corrosion of steel.

Molybdenum (Mo)—Introducing molybdenum as an alloy seems to exert unfavourable impacts on the MIC tendencies of low-alloy steels. Guo et al. [93] observed that the addition of 1.0 wt % Mo to low-alloy steel stimulated the formation of a biofilm by *P. aeruginosa* and subsequently resulted in pitting corrosion in the steel.

**Table 3:** Summary of studies on effects of alloying additions on MIC of steels.

Alloying Element	Steel Grade	Weight % of Alloying Element	Bacteria Used in Testing	MIC Susceptibility *	Ref.
Carbon (C)	K1878, K1063, S2684, K795, V2170, V2179	0.10, 0.25, 0.42, 0.61, 0.83, 0.94	*E. coli*	No effect	[72]
	1010, 1020, 1030, 1045	0.10, 0.17, 0.30, 0.51	*E. coli*	Negative effect	[67]
	A179, A516-70, A106-B	0.12, 0.08, 0.06	*D. desulfuricans*	Negative effect	[70]
	Q235, X65, X70, X80	0.18, 0.06, 0.06, 0.07	Mixed SRB culture separated from field samples	No effect	[71]
Copper (Cu)	API 5 LX	0.08-0.44	Natural consortia of bacteria and fungi	No effect	[76]
	Carbon steel, Corten steel	0.002, 0.29	*P. aeruginosa*	Positive effect	[77]
	X80 steel	1.06, 1.46, 2.00	*E. coli*, *S. aureus*, and SRB	Positive effect	[78]
	X80 steel	1.06, 2.00	SRB and *P. aeruginosa*	Positive effect	[79]
	BR5 mooring chain steel	0.0, 0.4, 0.8	Marine field exposure	Positive effect	[81]
	X80 steel	0.2, 1.06	SRB	Positive effect	[80]
	HSLA	0.01, 0.02, 1.34	*H. titanicae*	Positive effect	[82]
	AISI 8630	0.0, 0.4	*P. aeruginosa*	Positive effect	[83]
Sulfur (S)	AISI 8630	0.007, 0.018, 0.023	Tap water	Negative effect	[86]
	AISI 8630	0.007, 0.023	Cooling water	Negative effect	[84,85]
	AISI 8630	0.007-0.023	Natural consortia of bacteria and fungi	Negative effect	[76]
Cerium (Ce)	AISI 8630	0.0, 0.01	Tap water	Positive effect	[86]
	AISI 8630	0.015	Natural consortia of bacteria and fungi	Positive effect	[76]
	AISI 8630	0.0, 0.01, 0.015	Cooling water	Positive effect	[84]
Chromium (Cr)	Pipeline steel	<0.01, 0.57, 0.62, 4.62	*Desulfovibrio*	Positive effect	[92]
Nickel (Ni)	HSLA	4.78, 7.23	*H. titanicae*	Positive effect	[82]
Molybdenum (Mo)	Low carbon steel	0.0, 1.0	*P. aeruginosa*	Negative effect	[93]

* Negative effect, MIC susceptibility increases with alloying addition; Positive effect, MIC susceptibility decreases with alloying addition.

### 3.2. Steel Grain Size and MIC

#### 3.2.1. What Are Grains and Grain Boundaries?

**Figure 2:**
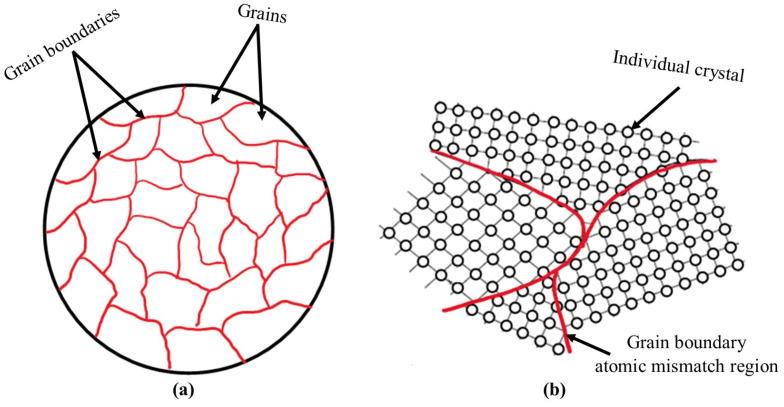
Schematic diagram showing grains and grain boundaries at (**a**) microscopic and (**b**) atomic levels.

Metals and alloys are generally polycrystalline materials [94,95]; the small crystals forming the microstructure are referred to as grains. The grains of metals and alloys are defined as the region with a consistent crystal lattice orientation (Figure 2a). Grain boundaries are the interfaces between adjacent grains where the atomic arrangements change (Figure 2b). The grain size and grain boundary characteristics are important microstructural features that can significantly influence the mechanical and corrosion properties of metals and alloys. [96]. Understanding grains and grain boundaries is essential for optimizing the performance of metallic materials in various applications, including MIC.

#### 3.2.2. Factors Affecting Grain Size of Steel

There are several processing variables that can affect the grain size of steel. These variables include the cooling rate during solidification [95,97,98,99], the amount of deformation during processing, and the addition of alloying elements [97,100].

**Figure 3:**
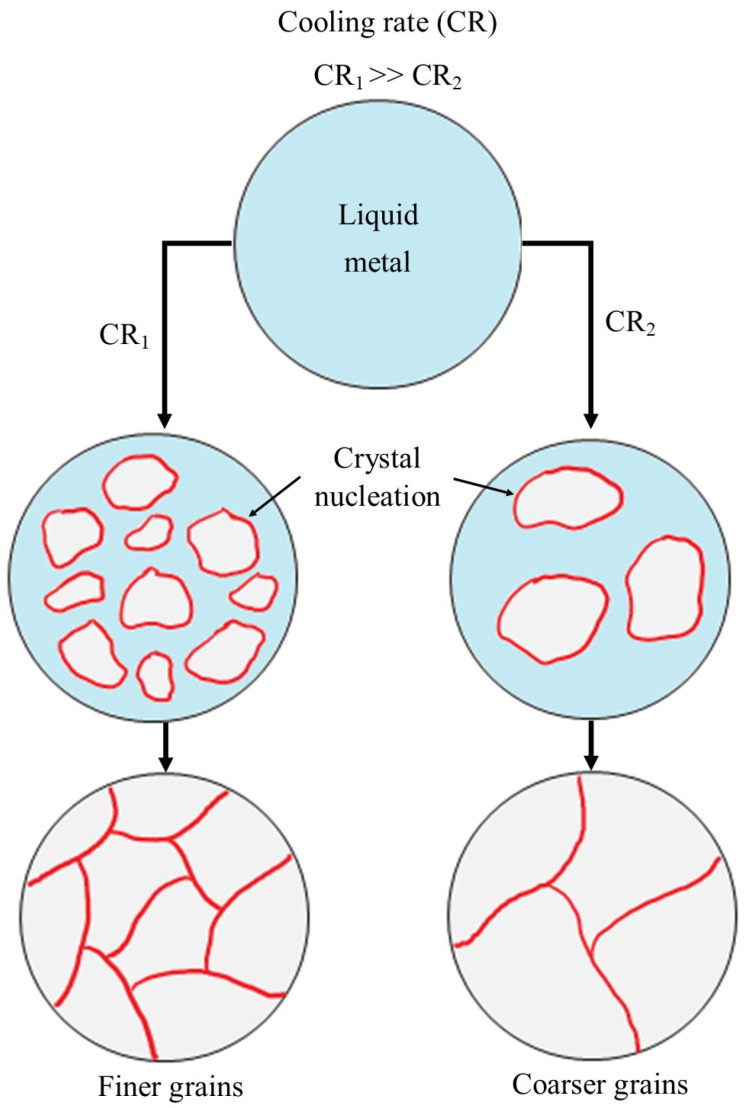
Schematic diagram showing effect of cooling rate on grain size.

As steel solidifies from a molten state, its microstructure, including grain size, is determined by the cooling rate (see Figure 3). Slower cooling allows atoms to form large crystals, resulting in a coarse-grained microstructure. Rapid cooling, on the other hand, limits the time for atoms to rearrange and form crystals, promoting smaller grains and a fine-grained microstructure. Additionally, it is noteworthy that metals and alloys can undergo post-manufacturing processes such as heat treatment or mechanical deformation to alter their grain size and grain boundary character. As such, post-manufacturing processes could potentially be effective strategies to aid in reducing MIC.

**Figure 4:**
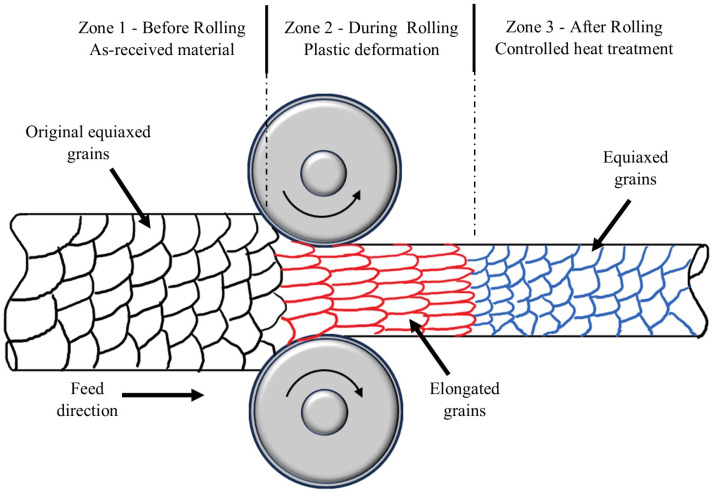
Schematic diagram showing effects of deformation and post-deformation heat treatment on grain size.

Mechanical deformation processes often used in the manufacturing process can have a notable effect on the grain size of steels [98,101]. When steel is subjected to mechanical forces that cause plastic deformation in bulk forming, such as rolling, forging, or drawing, the arrangement of atoms within the metal is altered, leading to changes in its microstructure, including the grain size. In certain deformation processes, such as rolling or drawing, the metal can experience elongation in one direction (Figure 4). This elongation can cause the grains to align and stretch in the direction of the applied force, resulting in an elongated grain structure developing anisotropic properties. Heat treatment is usually applied after the deformation processes to restore the elongated grains to equiaxed grains and to modify the grain size as required for specific applications, known as an annealing process. This involves recovery, recrystallisation, and grain growth. 

Alloying elements can also play a role in controlling grain size, as they can promote the formation of certain grain boundaries that inhibit grain growth [98,102]. Aluminium in is added to steel melts in small amount to control the grain size in steel [103,104,105]. During solidification, aluminium combines with oxygen and nitrogen to form aluminium oxide and aluminium nitride particles. These particles act as nucleation sites for the formation of fine grains, leading to a more refined grain structure in the final steel product.

#### 3.2.3. Grain Size and MIC

Grain size significantly affects the initial bacterial attachment and MIC of various metals and alloys [106,107,108]. Understanding this relationship is crucial in designing materials with enhanced resistance to bacterial attachment and MIC. While extensive research has been undertaken on the interplay between initial bacterial cell attachment and grain size/grain boundaries for stainless steels [106,109,110], there is a need to further explore the impact of grain size on bacterial attachment and MIC in other materials. This section specifically investigates the influence of grain size on bacterial attachment and MIC in carbon steels.

The role of carbon steel grain size and grain boundaries in the initial attachment of *E. coli* bacteria and subsequent MIC was investigated by Javed et al. [107]. The study involved the use of two distinct grain size structures of the same steel alloy (1010 carbon steel) achieved through heat treatment: one with an average grain size of 10 µm and the other with an average grain size of 50 µm. The results revealed that within the first 60 min, the initial bacterial attachment predominantly occurred along grain boundaries, with notably higher attachment observed on the small-grain size steel compared to the large-grain size steel. Furthermore, a distinct pattern of bacterial attachment was observed on the small- and large-grain steel specimens, with a more uniform distribution of bacterial attachment on the small-grain steel coupons, whereas bacterial aggregates were found on the large-grain steel coupons. The observed differences in the spatial distribution of the initial bacterial attachment could possibly be attributed to the segregation of impurities in the large-grain, heat-treated steel coupons, which occurs due to rapid cooling of the metal sample from a relatively high temperature. The formation of an MIC biofilm consistently begins with bacterial adhesion to a metal substrate, a process shaped by the interplay of attractive and repulsive forces between the microbial cells and the substrate. While the detailed mechanics of this interaction are not fully understood, they are believed to involve hydrophobic–hydrophilic interactions, van der Waals forces, or a combination of both [111,112]. Factors such as grain boundaries and the presence of impurities are thought to influence these interactions, potentially creating preferred sites for biofilm attachment and leading to uneven biofilm development.

In a separate investigation, Javed et al. [113] explored the relationship between initial sulfate-reducing bacterial attachment and the microstructure of 1010 carbon steel in modified Baar’s medium. The correlation was examined after 60 min of immersion by overlaying images of bacterial attachment onto the underlying metal microstructure at the same location. The study revealed that approximately 75% of the initial bacterial attachment occurred on the grain boundaries of the steel substrate.

Recently, Liu et al. [114] investigated the impact of grain size and crystallographic orientation on MIC of carbon steel in artificial seawater in the presence of SRB strain *Desulfopila corrodens*. The study showed that low-carbon steel AH36 with a coarsened grain structure exhibited a higher SRB cell attachment rate during the initial MIC phase and that more “defective” biofilms may evolve upon the materials, leading to a higher MIC rate. They also found anisotropic MIC behaviour in the material, with the <100> crystal directions showing the lowest dissolution rate within the body-centred cubic (BCC) structure of the low-carbon steel.

Franklin et al. [115] indicated that bacterial activity plays a crucial role in the initiation and propagation of corrosion pits on carbon steel surfaces in phosphate-containing electrolytes. Their research used scanning vibrating electrode (SVE) technology to observe the formation and inactivation of anodic and cathodic sites on a steel surface. The study found that under sterile, continuously aerated conditions, pits on the steel surface would initiate, then re-passivate. However, when aeration was absent, pits initiated and propagated, leading to corrosion. Interestingly, in the presence of a heterotrophic bacterium, which was isolated from a corrosion tubercle on a steel pipe in a freshwater environment, pit propagation was also observed, even under aerated conditions. Autoradiography of bacteria using ^14^C-acetate revealed that the sites of bacterial activity coincided with the sites of anodic activity on the steel surface. This suggests that bacteria preferentially attach to the corrosion products formed over corrosion pits, potentially creating stagnant conditions that promote further pit propagation. They concluded that bacterial cells are attracted to anodic regions either as they form or once established and that, once associated with an anodic region, re-passivation of the pit is unlikely, leading to continued corrosion propagation.

Several potential reasons for preferential bacterial attachment on grain boundaries have been proposed, including the following:

(i) Relatively higher surface energy of grain boundaries compared to grains caused by the high atomic mismatch in the grain boundary region [94,96], resulting in preferential attachment on grain boundary regions compared to the grains [106,116];
(ii) Preferential corrosion of grain boundaries compared to grains because of their low-equilibrium corrosion potential compared to the grains [94,96], attracting bacterial species via chemotaxis and/or surface charge [115,117,118]; and
(iii) The difference in elemental composition between the grain and grain boundaries, which may be responsible for preferential bacterial attachment [84,110].

Longer-term MIC studies have also consistently revealed correlations between the grain size of tested carbon steel and the extent of corrosion. For instance, Almahamedh et al. [119] investigated the MIC susceptibility of two carbon steel types commonly used in pipelines, namely API X52 and API X70. Their findings indicated that API X70 steel experienced more significant MIC attack than API X52, which was attributed to differences in grain size. API X52 steel has a relatively larger grain microstructure compared to API X70. It is crucial to note that both API X52 and X70 steels also differ in chemical composition, posing a challenge in attributing the observed difference in MIC attack solely to microstructural variations. Similar findings were reported by Javed et al. [107] in a study on 1010 carbon steel, examining its susceptibility to MIC in the presence of *E. coli* and in an M9 minimal-salt medium. They found that steel with a smaller grain size (average 10 µm) developed a thicker biofilm and experienced more severe MIC compared to steel with a larger grain size (average 50 µm).

Liu et al. [114] explored the relationship between the microstructure of AH36 low-carbon steel and MIC in the presence of *D. corrodens* in artificial seawater. Their investigation revealed that the same steel, featuring larger grains achieved through heat treatment, exhibited lower MIC resistance compared to steel with smaller grains. The researchers tentatively attributed this observation to the influence of grain orientations within the steel substrate and suggested that these assertions warrant further confirmation in future research.

In summary, both grain size and grain boundaries have been shown to play pivotal roles in influencing bacterial attachment and the occurrence/extent of MIC. It is important to note that these effects may vary depending on the specific microbe and type of steels involved. Therefore, caution should be taken when extrapolating results from previous studies involving one microbe to predict outcomes with other microbial species or different types of steel.

### 3.3. Steel Microstructural Phases and MIC

#### 3.3.1. What Is a Microstructural Phase?

Steel is an iron–carbon alloy that can have different microstructures, among which the most common are ferrite, cementite, austenite, martensite, pearlite, and bainite. A microstructure can consist of two or more phases, which are distinct components of the system that are separated by boundary surfaces. The microstructural phases present in an alloy are identified based on the composition and are affected by processing conditions; they play a crucial role in determining the physical, mechanical, and corrosion properties of steel [120,121,122].

**Figure 5:**
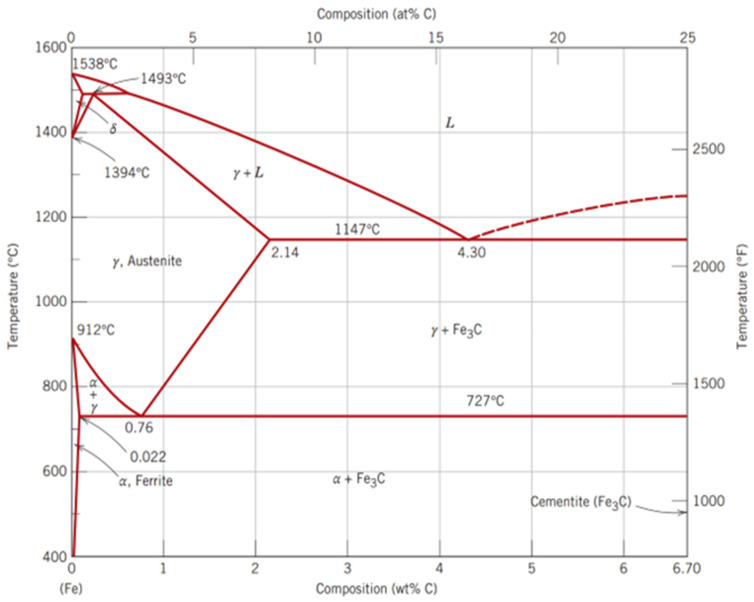
Iron–carbon phase diagram [96].

The iron–carbon equilibrium phase diagram shown as a function of carbon content and temperature in Figure 5 is an essential tool to understand and predict the behaviour of steel [123,124]. The formation and relative proportion of different phases depend on several factors, mainly chemical composition (carbon content and alloying elements), mechanical processing, heat treatments, and cooling rates [9,125]. The common microstructural phases found in steel are discussed below, along with their chemical compositions.

Ferrite (α)—It is a body-centred cubic (BCC) crystal structure characterized by its low carbon content, which typically ranges from 0.002% to 0.025%. It is the softest and most ductile phase of steel. Ferrite is commonly found in low-carbon steels, and its mechanical properties are greatly affected by its grain size, which can be controlled during the cooling of austenite below the critical point (912 °C at 0% C).

Cementite (Fe_3_C)—It is also known as iron carbide, has a complex orthorhombic crystal structure formed from iron and carbon. It is a wear-resistant, extremely hard, and brittle phase of steel, making it an important component of high-strength steels.

Austenite (γ)—It is a face-centred cubic (FCC) crystal structure formed when steel is heated above its critical temperature (typically 912 °C), which can vary depending on the carbon content. Austenite is a high-temperature phase of steel, and it is characterized by high ductility, softness, and toughness. It is commonly found in high-carbon and alloy steels. It is important to note that at temperatures below the critical temperature, austenite transforms into other phases, such as martensite or pearlite, each with distinct mechanical and corrosion properties.

Martensite—It is a nonequilibrium, body-centred tetragonal (BCT) crystal structure that forms when austenite is rapidly cooled by quenching in water or oil. Martensite formation involves a diffusionless transformation that leads to a high concentration of carbon and the presence of residual stresses. This phase is characterized by high hardness, strength, and brittleness. The rate of cooling and the temperature range are critical factors in the formation of martensite. Martensite transformation requires a rapid cooling rate, typically on the order of 10 to 1000 °C per second, depending on the composition of the steel [126]. The specific temperature range for martensite formation depends on the alloy composition, but in general, it occurs when steel is cooled from above its critical temperature to below the martensite start temperature. This temperature range can vary but is typically between about 200 °C and 500 °C, with lower temperatures resulting in higher hardness and greater brittleness of the martensitic steel [127]. 

Pearlite—It is a lamellar, two-phase microstructure that consists of alternating layers of ferrite and cementite. It is formed when austenite is slowly cooled down, and it is commonly found in medium- to high-carbon steels. Pearlite is known for its high strength, hardness, and wear resistance.

Bainite—It is a two-phase microstructure consisting of ferrite and cementite. It can form when steel is isothermally transformed at relatively low temperatures and is characterized by a fine and elongated structure that ensures high strength, toughness, and resistance to crack propagation.

#### 3.3.2. What Factors Affect Phase Formation?

While the content of carbon in steel can be broadly modulated to obtain specific mechanical properties, it is not the only element to consider. In fact, the presence of other alloying elements can radically modify the iron–carbon phase diagram to an extent that depends on the type of elements and their concentration, as they can stabilize or promote the formation of a specific phase over the other [102,128].

Heat treatments such as annealing, quenching, and tempering can significantly affect the microstructure and resulting phase formation of steels. For example, annealing at high temperatures can promote the formation of austenite, whereas quenching can promote the formation of martensite. The rate of cooling during the heat treatment can also affect the resulting microstructure, with fast, moderate, and slow cooling rates promoting the formation of martensite, bainite, and pearlite, respectively [95].

Finally, mechanical processing, such as hot and cold working, can greatly impact the steel microstructure. Hot working, which is performed at elevated temperatures, can lead to the formation of coarse-grained microstructures, whereas cold working, which is performed at room temperature, can lead to the formation of fine-grained microstructures. Moreover, both hot and cold working can promote the formation of deformation-induced microstructures, such as dislocations and twins, thus affecting the mechanical properties of steels [129,130,131].

If, on one side, the complex interaction between these influencing factors can make the prediction of the microstructure and phase formation of steels challenging, on the other side, it can provide opportunities to tailor the microstructure to achieve desired properties and performances.

#### 3.3.3. Microstructural Phases and MIC

Microorganisms adhere to metal surfaces and subsequently colonize and proliferate, forming biofilms. The development of these biofilms is influenced by the microstructural features of the metal surface [87,132,133]. The effects of factors such as microstructural phases, grain boundaries, residual stresses, surface chemistry, and the presence of alloying elements on the onset and progression of MIC have been investigated and assessed in the literature [67,107,109,110,134,135,136].

The relationship between metal composition and MIC is dependent on the following two factors: microstructural phases and alloying elements/impurities [110,137]. The effect of alloying elements on MIC of steel was discussed in detailed in Section 3.1.4. Javed et al. [67] examined the impact of composition and microstructure in various grades of carbon steel on the initial attachment (within 60 min) of *E. coli* and their subsequent influence on corrosion over a longer term (28 days). All experiments used a medium consisting of essential salts and glucose, which served as an energy source for bacterial growth. Four different carbon steel grades, namely 1010, 1020, 1030, and 1045, were evaluated, each with increasing pearlite contents. The study revealed that initial bacterial attachment increased over time across all grades of carbon steel. However, the rate and magnitude of bacterial attachment varied among the different steel grades, with significantly lower attachment observed on steels with higher pearlite-phase content. This variability in bacterial cell attachment was attributed to the differing ferrite-to-pearlite phase ratios in the distinct grades of steel, leading to varying micro-galvanic cell potentials among these phases. 

Studies investigating the reactions of different phases to MIC attack concluded that ferrite and austenite are more susceptible to this type of corrosion. Stein [138] suggested that when subjected to oxidizing media, the austenite phase present in the ferrous alloys will be more vulnerable to microbial attack. When the corroding medium is reducing, instead, the ferrite phase is preferentially corroded. Similar results demonstrating the preferential corrosion of austenite and ferrite were presented by Damborenea et al. [88] and by Borenstein [134,139], respectively.

### 3.4. Inclusions in Steel and MIC

#### 3.4.1. Types of Inclusions in Steels and Their Effects on the Abiotic Corrosion of Steels

Inclusions in steel can be defined as non-metallic compounds formed during production and processing that do not incorporate into the molecular structure of the alloy. The types of inclusions in carbon and low alloy steels are influenced by various factors, including casting techniques and post processing [140,141,142,143]. Based on the source of origin, inclusions can be classified into endogenous and exogenous types [142,144].

Endogenous inclusions are formed during the deoxidation process or precipitated during solidification of the steel [142,143]. During steel transfer from the furnace to the ladle, tundish, mould, or continuous caster, air pickup is nearly unavoidable, leading to typical endogenous inclusions such as alumina (Al_2_O_3_) or silica (SiO_2_) [142,143]. Precipitated inclusions, on the other hand, occur during solidification, with elements like alumina, silica, aluminium nitrides, and titanium nitrides, and sulfides precipitating from the molten steel [142,144,145,146]. If rare-earth elements are added to the ladle, rare-earth oxides will also form [147,148].

Exogenous inclusions due to reoxidation are formed when the molten metal comes into contact with air during pouring, either from the bath to the ladle or from the tundish to the mould or continuous caster [140,149,150]. Exogenous inclusions tend to be larger than normal deoxidation inclusions due to preferential reactions with elements like Al, Ca, Mg, and Si [149,150,151,152,153,154,155]. Compared to the smaller indigenous inclusions, exogenous inclusions are fewer in number but can act as stress risers, impacting mechanical properties such as fatigue and ductility [141,144,149,150].

Inclusions in steel can significantly impact the physical properties by inducing localized abiotic corrosion. Such abiotic corrosion can be categorised into thermodynamic instability and anisotropy between the inclusions and the matrix [143,155]. Thermodynamic instability occurs when inclusions are more thermodynamically unstable than the surrounding matrix, making them more susceptible to corrosion. For example, MnS inclusions are common in various steels and are thermodynamically unstable in the presence of oxygen, leading to the formation of pits and cracks around them, ultimately resulting in localized corrosion [143,144,152,156].

Anisotropy between inclusions and the matrix occurs when inclusions possess different electrical or chemical properties than the surrounding matrix [143]. Many inclusions, such as aluminium oxides or nitrides, tend to be more cathodic than the steel matrix, making them more prone to corrosion and leading to localized galvanic-coupling effects [143,152]. As discussed in reference [157], there has been an understanding of the potential effect of inclusions on the abiotic corrosion of steel since the start of the 20th century. Stainless steels have been the focus of a lot of the work on inclusions and corrosion [158,159], although some notable examples of work looking at inclusions and abiotic corrosion of carbon steels can be found in references [160,161,162].

#### 3.4.2. Inclusions in Carbon Steel and MIC

While there have been various reports on the potential effects of inclusions and abiotic corrosion of carbon steels, there only a relatively small number of papers discuss how inclusions may affect/relate to MIC. The reports can largely be split into the following two categories: anecdotal evidence (e.g., references [163,164,165,166]) and more detailed laboratory studies. Anecdotal links between MIC of steels and the presence of inclusions have been discussed in field failure reports or tests with limited evidence to definitively verify whether inclusions were actually the cause of the observed corrosion. The latter could result from either a lack of appropriate controls or the fact that the tests did not include detailed experiments specifically designed to study the effect of inclusions, so were somewhat more correlation- than causation-focused in nature.

Some of the key observations from studies of inclusions and MIC include the following:

(i) Evidence of increased localized corrosion of the bulk metal matrix surrounding inclusions when tested under biotic conditions (e.g., references [167,168,169]);
(ii) The effects of the shape, size, and orientation of inclusions relative to the surface being tested on the extent to which microorganisms will interact with inclusions and the extent of subsequent localized corrosion. Additives such as cerium can change the dimensions of MnS inclusions and, hence, the extent of localized MIC attack [84,87];
(iii) Relatively short-time-frame (i.e., less than 48 h) observations, indicating that inclusions may be a preferred site for microbial attachment [84,85].

As discussed earlier, methods to examine the correlation between inclusions and MIC are not necessarily straightforward. Hence, it is worth mentioning a few examples of techniques that could be used and some of the things that researchers need to be careful of. One interesting method to test whether inclusions are affecting the outcome of an MIC test is to remove any inclusions present on the surface of control samples prior to testing using sulfuric acid [170]. Another paper of note is that by Stipaničev et al. [171], which proposes the use of etched cross-sections of test samples as a way of determining if the microstructure or inclusions are related to localized corrosion. Researchers should note that the size and shape of inclusions present on a surface can depend on the orientation of the sample relative to the rolling direction [168] and that this may affect the outcomes of their testing. Finally, it is worth noting that one needs to be extremely careful of the cleaning processes used to remove corrosion products. Many cleaning processes use dilute acids, which can potentially lead to preferential dissolution of inclusions, which could be incorrectly interpreted as localized corrosion due to MIC [172,173].

### 3.5. Steel Welding and MIC

#### 3.5.1. What Is Welding?

Carbon steels are commonly used in structural applications, which often require joining by welding to realise complex structures. Consequently, the integrity of the structure will depend on the strength of both the steel and any welded joints. Welding can be defined as a manufacturing process whereby two or more similar or dis-similar materials are joined together permanently, forming coalescence with or without the application of heat, filler material, or external pressure. The welding process is widely employed in a variety of outdoor and indoor environments, underwater, and even in outer space [174,175,176]. There are two main categories of welding methods, namely fusion welding [177,178] and solid-state welding (SSW), also called pressure welding [179].

Fusion welding is a process that uses heat to join two or more materials by heating them to their melting point. This method does not usually require external pressure, except for processes like resistance welding, where substantial contact pressure is needed for a secure joint. Fillers may be used, but they are not always necessary. It includes gas welding [180,181,182], arc welding [183,184,185], resistance welding [186], and intense-energy beam welding [187]. The choice of method depends on the materials being joined and the desired properties of the welded joint. Despite being applied in several industrial sectors, fusion welding has numerous drawbacks, such as the development of internal stresses, distortions, microstructural changes, and intermetallic compounds in the welded region, and harmful effects such as flashlights, ultra-violet radiation, high temperatures, and fumes [188,189]. In addition, especially when dealing with dissimilar joints, the selection of filler metals and the differences in melting points, mechanical properties, and thermal expansion of the base metals need to be taken into account when applying fusion welding [175,176,178].

In order to overcome the aforementioned issues with fusion welding and to fulfil new design demands in accordance with material advancements, solid-state welding (SSW) was developed. SSW involves joining two materials in the solid-state phase without melting them. Instead, it relies on high pressure and/or high heat to create a bond between the materials at the atomic level, though never exceeding the melting points of the base components [190]. The main techniques of SSW are diffusion bonding [191], friction stir welding [192,193,194,195], forge welding [196,197,198], cold welding [199,200,201], ultrasonic welding [202,203], roll welding [202,204], and explosion welding [205,206]. This technique allows for the production of strong, high-quality joints without the need for filler materials and can be used to join dissimilar metals that cannot be welded using conventional methods.

#### 3.5.2. How Welding Treatments Affect Microstructural Variation

**Figure 6:**
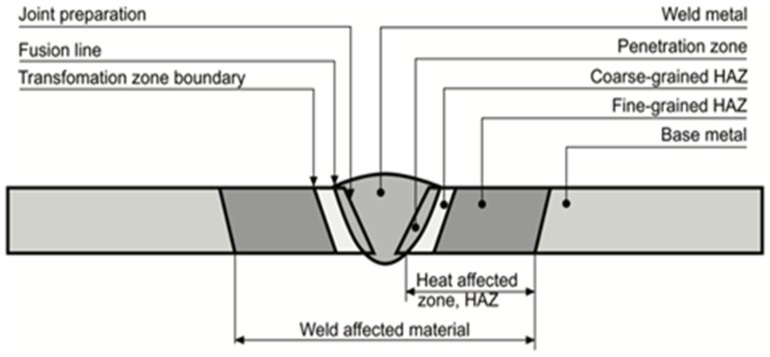
Different zones and boundaries in welded metal [176].

The properties of the original base metal after welding are largely determined by the type of filler and base material and by the weld method and process parameters used, while the properties of the adjacent zone, called the heat-affected zone (HAZ), depend on the base material composition and the thermal energy released in the welding process. The different areas of a welded metal joint are shown in Figure 6 and explained in the following paragraph.

**Figure 7:**
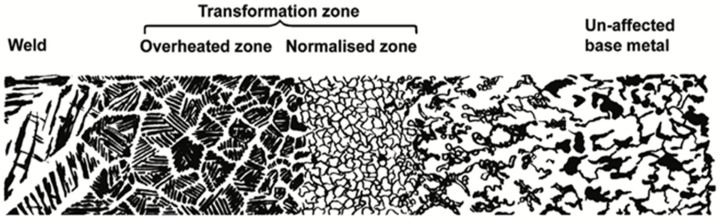
Influence of the welding process on the base metal microstructure [176].

Carbon steel, which usually has a ferritic–pearlitic structure, is often joined by welding. Figure 7 illustrates the effect of the weld heat input on the base material microstructure, where the following three main zones can be identified: the weld zone, the transformation/heat-affected zone (HAZ), and the unaffected base material. The weld zone can have tailored properties that meet specific performance requirements thanks to an appropriate selection of the filler metal and welding parameters. In the case of single-pass welds, it can have a Widmanstätten structure, characterized by a distinct pattern of elongated interlocking plates or needles that are arranged in a specific crystallographic direction. A multi-pass weld, on the contrary, will generally display a normalized weld structure, in which each weld bead will be heat-treated by the consequent bead. The region adjacent to the weld is the HAZ, which consists of the following two parts: the normalized zone, which has been heated slightly above the A3 line in the iron–carbon diagram (Figure 5), and the overheated zone, which has been subjected to temperatures significantly higher than A3, up to the melting point of the material, and generally shows enlarged grains and Widmanstätten orientation. Between the normalized zone and the unaffected metal, there is the so-called structural change zone, which usually reaches temperatures between A1 and A3.

Among these regions, the HAZ is of particular concern because it can affect the mechanical properties of the weld joint—in particular, its toughness and resistance to cracking. As expected by looking at the iron–carbon diagram in Figure 5, the high temperatures reached during welding induce the formation of austenite, which, during cooling, will turn into a different structure, depending on the cooling rate. A very high cooling rate will impede the formation of ferrite and pearlite, favouring the creation of martensite, which is very hard and brittle, consequently increasing the risk of hydrogen embrittlement and cracking. Indeed, hydrogen can be released into the weld metal and diffuse to the base material, decreasing its solubility during cooling and causing cracks.

Low-carbon steels have low hardenability and can usually weld easily. The welded regions exhibit a higher strength than the base metal due to two main factors, namely the finer pearlite microstructure that generates during the cooling of the HAZ and the presence of retained austenite along the ferrite grain boundaries, which limits recrystallization and preserves a fine grain size. On the contrary, medium- and high-carbon steels can result in poor toughness of the weldment due to the formation of martensite in the HAZ. To solve this issue, it is common to resort to preheating of the material to slow down the cooling rate or to post heating to temper any formed martensite [120,176]. 

#### 3.5.3. What Are Pre- and Post-Weld Treatments?

The localized heating resulting from welding can be responsible for potential stresses and distortions, with consequent failure. The solutions implemented to deal with this issue can be mechanical (appropriate design, presetting/offsetting, mechanical restraints, or mechanical stress relief) or thermal (preheating, limiting the heat input and control of other weld parameters, thermal tensioning and heat-sink welding, sequential welding, or flame-straightening/heat treatments) [174,207].

During the process of presetting/offsetting, the pieces are positioned at a predetermined angle, and as the weld cools and contracts, it causes them to shift into the correct alignment without restraining them, thus minimizing the residual stresses. Mechanical restraints like jigs and clamps decrease the level of distortion by keeping the component in position but can increase residual stresses. Mechanical stress relief techniques consist of the redistribution of residual stresses by using ultrasound or shock peening, which employs a high-density, short-pulse laser to alter the surface and subsurface components, resulting in compressive residual stresses that enhance its damage tolerance.

Residual stresses caused by welding can be reduced by preheating of the parts, limiting the heat input during welding, and controlling weld parameters like the travel speed and the number of weld passes [208]. Thermal tensioning consists of moving the heat source on the weld torch, regulating the heating and cooling rates of the weld, and controlling distortions and residual stresses. The same process with a cooling source is called heat-sinking welding. Sequential welding refers to a welding technique like balanced welding, back-skip welding, or back-step welding that enables sections of the component to move during the process. Flame straightening distortions to be straightened out by providing intense, localized heat through a heating torch. Finally, proper stress relief can also be provided through post-weld heat treatments (like the use of heated jackets around the components).

#### 3.5.4. Welds and MIC

The rapid attack of weld regions with through-thickness penetration that occur in the timescale of months is one of the most widely reported failure modes for MIC. These failures often include a small pinhole on the surface with a large cavity (often described as having an ink-bottle shape) in the weld region underneath [209,210,211,212,213]. Reports on this type of failure, however, are heavily dominated by cases involving stainless steels, such as the 304 and 316 grades.

For work on stainless steels, researchers have suggested a number of potential causes of rapid failure of welds due to MIC, including the following:

(i) Microstructural effects [87,106];
(ii) Compositional effects [214]; and
(iii) Surface roughness [106,215].

Post treatments of welds, including annealing and avoiding/removal of heat-tinted scale (e.g., gas shielding during welding and pickling), have been suggested as ways to reduce these problems [209,210,216,217,218]. However, there can be some practical difficulties in implementing these measures [218,219].

Work on and reports of the MIC of carbon and low-alloy steel welds are definitely less comprehensive and less conclusive when compared to the work published on stainless-steel welds [84,85,108,220,221,222,223,224,225,226,227]. In addition, a wide variety of test configurations have been used, making it very difficult to compare results or determine trends. Test media range from natural seawater [220,221,225] to microbiological growth media containing SRB [222,223,224,226]. Various types of welded sample configurations have been used in research studies, including samples that encompass complete weld samples, i.e., the base metal, the HAZ, and the weld in one sample. Additionally, samples of individual sections/zones of the weld or samples that have been heat treated to simulate weld regions have been employed. Some of these samples were polished before testing, while others were left unpolished. Although there are potential advantages to each of these different test configurations, it makes comparisons very challenging. The MIC test results are split roughly in half between those that indicate increased corrosion of weld regions [84,85,220,221,225] and those that show that the base metal has greater levels of pitting attack when compared to the HAZ and weld zone [222,223,224,226]. Tests on microbiological attachment also show mixed results, with some tests indicating increased microbial attachment in the weld zone [84,85,226], while others showed greater attachment on the base metal [221,223]. One notable paper on microbial attachment is the work by Liduino et al. [221], who reported more bacteria attached on unpolished welds (with suggestions that this was due to surface roughness), while polished welds had similar numbers of bacteria as the base material.

Some key findings, comments, and suggestions from this review include the following:

(i) Work needs to be undertaken to clarify the effects of the different testing arrangements used for weld and MIC studies on the obtained results.
(ii) Only limited work has been undertaken to investigate the effects of different welding processes and materials on subsequent MIC.
(iii) While testing separate sections of specific regions of welds or simulated regions produced by heat treatments is understandable, do these individual samples behave the same as “complete” samples containing all the weld zones?
(iv) Should welds be polished before lab testing or joins as per the industry standards to be used?
(v) Should there be a minimum duration of testing required before meaningful results can be obtained?
(vi) There is a need to make sure that appropriate abiotic controls are included in the testing.

## 4. Future Perspectives

Considering the multidisciplinary nature of MIC, which encompasses microbiology, chemistry, and metallurgy, future research should attempt to integrate an understanding of all these aspects, focusing on how metallurgical factors, environmental conditions, and diverse microbial species interact. Understanding these interactions becomes even more critical as infrastructure is increasingly being deployed into novel ecosystems like the deep sea, where extreme conditions can unpredictably alter MIC processes [228]. Notably, these environments are highly sensitive to traditional MIC prevention strategies, such as coatings and biocides. Therefore, a detailed understanding of the role of metallurgical and microstructural design is essential. Such research could pave the way for the development of new varieties of carbon steel specifically engineered to be more resistant to MIC and, therefore, better-suited for these extreme and delicate environments. To achieve this, systematic documentation and disclosure of the following key information is crucial:

*i. Composition of the steel substrate:* Beyond acknowledging the use of carbon steel in a test, it is essential to provide information about the specific alloy type of carbon steel being used and its chemical composition. Even minor quantities of alloying elements can significantly influence the susceptibility of the steel to MIC.

*ii. Microstructure of the steel substrate:* The microstructure of the steel substrate is crucial in MIC. Different microstructural phases may exhibit varying levels of susceptibility to microbial attack. Understanding the microstructure allows researchers to correlate observed corrosion behaviour with specific phases or regions within the steel.

*iii. Working history of the steel substrate:* The working history of the steel, including mechanical working and heat treatment, plays a pivotal role in determining its susceptibility to MIC. Documenting the working history allows researchers to comprehend the influence of processing conditions on the microstructure, mechanical properties, and overall corrosion resistance of the steel.

*iv. Details of inclusions present in the steel substrate:* Inclusions within the steel matrix present potential sites for localized corrosion initiation. Providing information about the type, size, and distribution of inclusions is crucial for understanding the material’s heterogeneity. Techniques such as scanning electron microscopy (SEM) coupled with energy-dispersive X-ray spectroscopy (EDS) can be employed to characterize inclusions.

*v. Methods of post-MIC test cleaning treatment:* It is important to provide explicit descriptions of the methods employed for post-MIC test cleaning to guarantee consistency and enable comparison across different studies. This should include specifying whether techniques like chemical cleaning, mechanical brushing, or a combination of methods were used. It should be noted that chemical cleaning methods commonly used for the cleaning of steel substrates after MIC testing can result in the dissolution of inclusions present in steel. This dissolution can lead to the formation of localized material loss that is often mistakenly interpreted as pitting corrosion due to MIC. For a more in-depth understanding of the impact of cleaning methods on inclusion dissolution and MIC, additional information can be found in references [90,173].

Incorporating the above details into research on MIC of carbon steel enhances the comprehensiveness of the study, allowing for more accurate comparisons and a deeper understanding of the metallurgical factors influencing corrosion behaviour.

## 5. Conclusions

This review has summarised our current understanding of how specific metallurgical factors impact the occurrence of MIC in carbon steels. The main aim is to underscore the importance of the metallurgical factors for individuals who lack expertise in metallurgy or who are new to the field, particularly in the context of MIC.

Metallurgical factors often receive limited attention in MIC studies, which can tend to focus on microbiological and chemical aspects. However, a thorough understanding of the metallurgical properties of steel, such as its chemical composition, grain size, grain boundaries, microstructural phases, inclusions, and weldments, is vital. These factors play a substantial role in determining the vulnerability of steel to MIC. 

While our paper focuses on the metallurgical properties affecting MIC of carbon steel, we acknowledge the broader implications for understanding MIC in other metals. It is important for future research to also document and analyse these metallurgical attributes in studies of MIC in other metals. This approach can help identify common trends and principles, improving our understanding of MIC across different materials and aiding in the development of strategies to mitigate MIC in a wider range of metals.

In essence, a deeper insight into metallurgical principles not only increases our comprehension of MIC but also aids in the development of more effective prevention and mitigation strategies. Furthermore, it enhances the reliability of comparisons between different studies, promoting a more integrated and comprehensive approach to MIC research.

## Figures and Tables

**Table 1:** Examples of AISI/SAE and UNS designation systems used for carbon steels.

Alloy Type	AISI/SAE Designation	UNS Designation	Sub-Group Types	AISI/SAE Designation	UNS Designation
Carbon steel	1xxx	G1xxx0	Plain carbon steel, Mn 1% max	10xx	G10xx0
			Resulfurised free-cutting steel	11xx	G11xx0
			Resulfurised/rephosphorised free-cutting steel	12xx	G12xx0
			Plain carbon steel Mn 1–1.65%	15xx	G15xx0

**Table 2:** The effects of different alloying elements in carbon and low-alloy steels.

Element	% Commonly Used	Effect on Steel Properties
Carbon C	0.02% to 2.1%	Increases hardness, strength, and wear resistance. Higher carbon content also decreases ductility and weldability while increasing the risk of brittleness.
Manganese Mn	0.3% to 1.5%	Improves hardenability, tensile strength, and workability of steel. It can help counteract the harmful effects of sulfur impurities.
Silicon Si	0.08% to 2.0%	Used as a deoxidizer in steelmaking. It improves strength, hardness, and electrical conductivity. It also promotes resistance to oxidation and corrosion.
Sulfur S	0.05% to 0.15%	Normally considered an impurity. Can reduce toughness and ductility. In some cases, used to improve machinability.
Nickel Ni	8.0% to 10% in stainless steel	Commonly used in stainless-steel alloys. Enhances toughness, impact resistance, and corrosion resistance of steel.
Chromium Cr	≥10.5% and up to 18.0% in stainless steel	Widely used in stainless steel alloys. Improves corrosion resistance, wear resistance, and high-temperature strength.
Molybdenum Mo	0.2% to 5.0%	Increases the hardenability and strength of steel at high temperatures. Improves the toughness and corrosion resistance of stainless-steel alloys.
Vanadium V	Up to 0.15%	Enhances strength, wear resistance, and toughness. It refines grain size and promotes fine carbide formation, which contributes to increased strength and improved impact resistance.
Tungsten W	2% to 18%	Improves high-temperature strength and hardness. It is commonly used in tool steels to enhance wear resistance and toughness.
Copper Cu	0.1% to 0.4%	Improves corrosion resistance and enhances the atmospheric corrosion resistance of steel. It also increases the strength and electrical conductivity of certain steel alloys.
Aluminium Al	0.95% to 1.30%	Used as a deoxidizer and grain refiner in steel production. It helps control grain size and improves the steel’s strength and toughness.

## Data Availability

Not applicable.
